# The Yeast Histone Chaperone Hif1p Functions with RNA in Nucleosome Assembly

**DOI:** 10.1371/journal.pone.0100299

**Published:** 2014-07-29

**Authors:** Amy R. Knapp, Huanyu Wang, Mark R. Parthun

**Affiliations:** Department of Molecular and Cellular Biochemistry, The Ohio State University, Columbus, Ohio, United States of America; Southern Illinois University School of Medicine, United States of America

## Abstract

**Background:**

Hif1p is an H3/H4-specific histone chaperone that associates with the nuclear form of the Hat1p/Hat2p complex (NuB4 complex) in the yeast *Saccharomyces cerevisiae*. While not capable of depositing histones onto DNA on its own, Hif1p can act in conjunction with a yeast cytosolic extract to assemble nucleosomes onto a relaxed circular plasmid.

**Results:**

To identify the factor(s) that function with Hif1p to carry out chromatin assembly, multiple steps of column chromatography were carried out to fractionate the yeast cytosolic extract. Analysis of partially purified fractions indicated that Hif1p-dependent chromatin assembly activity resided in RNA rather than protein. Fractionation of isolated RNA indicated that the chromatin assembly activity did not simply purify with bulk RNA. In addition, the RNA-mediated chromatin assembly activity was blocked by mutations in the human homolog of Hif1p, sNASP, that prevent the association of this histone chaperone with histone H3 and H4 without altering its electrostatic properties.

**Conclusions:**

These results suggest that specific RNA species may function in concert with histone chaperones to assemble chromatin.

## Introduction

Our understanding of chromatin assembly has grown rapidly in the past several years. It is now apparent that in addition to the assembly of chromatin structure that occurs during DNA replication, which is known as replication-coupled chromatin assembly, a significant amount of chromatin assembly also occurs outside of S-phase and is known as replication-independent chromatin assembly (or histone exchange)[1]–[6]. A large number of factors have been identified as participating in chromatin assembly such as histone chaperones, histone modifying enzymes and ATP-dependent chromatin remodeling complexes.

While many proteins have been found to participate in chromatin assembly, it is likely that additional factors have yet to be identified. This is particularly apparent in yeast, where many of the proteins that are thought to play central roles in the histone deposition process are not essential for viability. For example, the CAF-1 and HIR complexes are histone H3/H4 chaperones that are integral components of the replication-coupled and replication-independent chromatin assembly pathways, respectively. Genes encoding the subunits of these complexes can be deleted both individually, and in combination, without a complete loss of viability[7]–[9]. Hence, there are clearly multiple pathways of chromatin assembly that can, at least partially, compensate in the absence of other pathways.

Hif1p, which shows sequence similarity to the N1/N2 family of histone chaperones, is a histone chaperone with specificity for histones H3 and H4[10]–[12]. Hif1p was originally identified as a subunit of the nuclear NuB4 histone acetyltransferase complex that also includes Hat1p and Hat2p[10], [13]. *In vivo*, Hif1p has been shown to be involved in the reassembly of chromatin structure that occurs following the recombinational repair of a DNA double strand break and some of its involvement in this process is independent of Hat1p[14]. In addition, Hif1p was also shown to be involved in replication-independent histone exchange[15]. However, *in vitro* experiments demonstrated that Hif1p is not capable of depositing histones onto DNA alone but can facilitate the deposition of histones on a closed circular plasmid in the presence of a yeast cytosolic extract[10]. In an effort to identify novel chromatin assembly factors, we have fractionated this extract to determine the factors that function with Hif1p in histone deposition. Surprisingly, our data suggest that histone chaperones can function in conjunction with RNA to accomplish chromatin assembly.

## Results

Recombinant Hif1p can specifically interact with histone H3/H4 complexes but is not capable of depositing the H3/H4 complexes onto DNA (as measured by a conventional plasmid supercoiling assay). However, the addition of a cytosolic extract allows rHif1p to facilitate histone deposition. This suggests that the cytosolic extract contains a factor (or factors) that function in conjunction with rHif1p in the deposition of histones.

### Hif1p functions with a cytosolic factor in histone deposition

**Figure 1:**
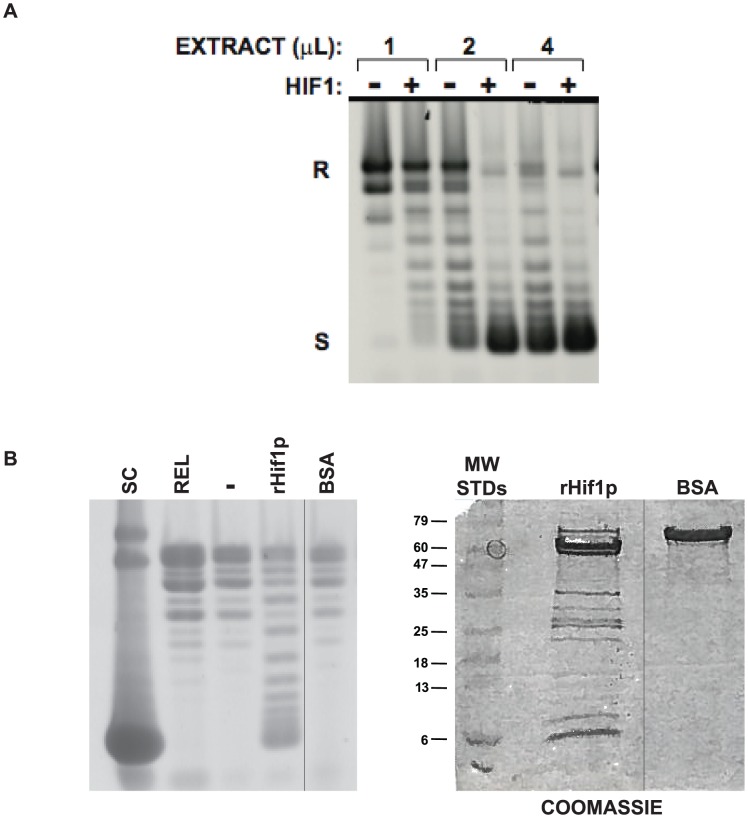
Concentration dependence of cytosolic extract-mediated chromatin assembly. A) Varying amounts of yeast cytosolic extract were incubated with core histones and a relaxed, circular plasmid for 1 hour in the presence or absence of rHif1p (as indicated). Following removal of protein, DNA was isolated and resolved by agarose gel electrophoresis and visualized by staining with Sybr Gold. Migration of relaxed (R) and supercoiled (S) forms of the plasmid are indicated. B) Yeast cytoplasmic extract was assayed for chromatin assembly as above with either rHif1p or BSA, as indicated (left panel). The amounts of rHif1p and BSA used in the chromatin assembly assay were resolved by SDS-PAGE and stained with coomassie blue (right panel). For both the left and right panels, all of the lanes were run on the same gel but an irrelevant lane was removed from the figure.

The cytosolic extract used in these experiments is generated from *hif1*Δ yeast spheroplasts that are osmotically disrupted in buffer with a low concentration of salt (50 mM NaCl). As seen in Figure 1A, the cytosolic extract was capable of assembling nucleosomes onto a relaxed circular plasmid as indicated by the introduction of supercoils into the plasmid. However, the addition of rHif1p significantly stimulated this activity. The effect of Hif1p on histone deposition was not a non-specific protein effect, as an equal concentration of BSA had no effect on histone deposition activity (Figure 1B).

The synergistic activity seen between Hif1p and the cytosolic extract was, in some ways, similar to the increased levels of chromatin assembly that are seen when Asf1p is assayed with either CAF-1 or the HIR complex[16]. Therefore, a candidate gene approach was used and cytosolic extracts were generated from strains in which potential chromatin assembly factors had been deleted. We found that extracts lacking Asf1p, Cac1p (largest subunit of CAF-1), Hir2p, Nap1p, Rtt106p, Hat1p, Hat2p or Chd1p showed no defects in chromatin assembly activity when combined with rHif1p, suggesting that the cytosolic extract might contain a novel chromatin assembly factor (and data not shown).

### Fractionation of cytosolic extracts to identify chromatin assembly factor(s)

**Figure 2:**
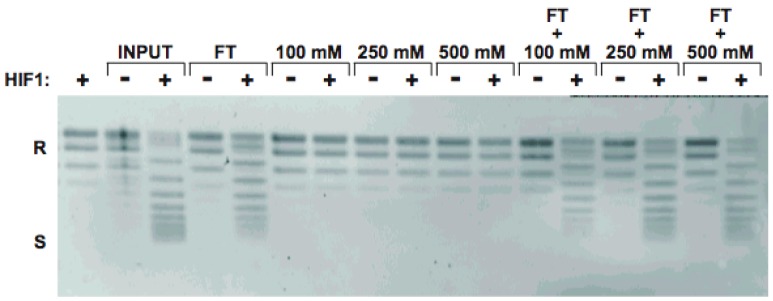
Fractionation of the chromatin assembly activity by anion exchange chromatography. A) Cytosolic extract was applied to a DEAE Sepharose column in buffer containing 50 mM NaCl. Proteins that did not bind to the column were collected as the flow-through fraction (FT). Column was then step eluted with buffers containing 100 mM, 250 mM and 500 mM NaCl, as indicated. Aliquots of each fraction were assayed for chromatin assembly activity by incubation with core histones and a relaxed, circular plasmid for 1 hour in the presence or absence of rHif1p (as indicated). Following removal of protein, DNA was isolated and resolved by agarose gel electrophoresis and visualized by staining with Sybr Gold. Migration of relaxed (R) and supercoiled (S) forms of the plasmid are indicated.

We used conventional protein purification techniques to isolate the cytosolic extract factor(s) biochemically. The cytosolic extract was loaded onto a DEAE Sepharose anion exchange column equilibrated in buffer containing 50 mM NaCl. Proteins that flowed through the column (FT) were collected and then the column was washed and successively step eluted with buffers containing 100 mM NaCl, 250 mM NaCl and 500mM NaCl. Hif1p-enhanced the chromatin assembly activity that was found in the FT fraction but not in any of the step elution fractions (Figure 2). Addition of the step elution fractions to the FT did not alter its activity suggesting that all of the factors necessary for the activity are in the FT fraction from the DEAE Sepharose column.

**Figure 3:**
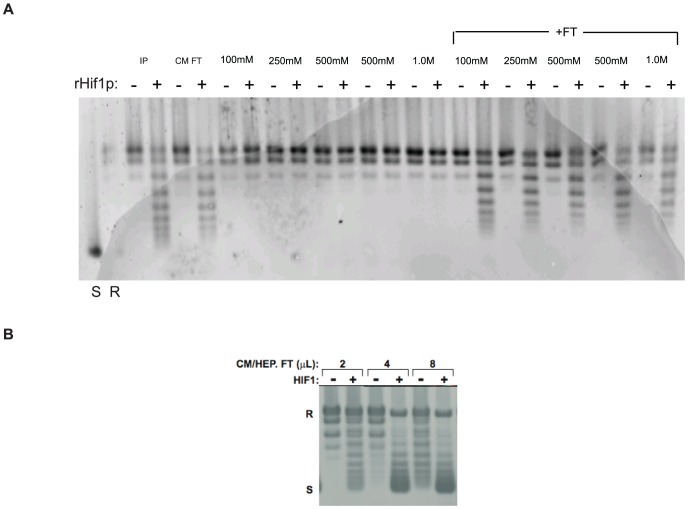
Further fractionation of the chromatin assembly activity. A) The FT fraction from the DEAE sepharose column was applied to a carboxymethyl Sepharose column in buffer containing 50 mM NaCl. Proteins that did not bind to the column were collected s the flow-through fraction (FT). Column was then step eluted with buffers containing 100 mM, 250 mM, 500 mM NaCl and 1.0 M NaCl as indicated. Aliquots of each fraction were assayed for chromatin assembly activity by incubation with core histones and a relaxed, circular plasmid for 1 hour in the presence or absence of rHif1p (as indicated). Following removal of protein, DNA was isolated and resolved by agarose gel electrophoresis and visualized by staining with Sybr Gold. B) CM sepharose and heparin sepharose columns run in series. The fraction that flows through both columns was assayed for chromatin assembly activity as described above.

As the chromatin assembly activity flowed through a DEAE Sepharose column at low salt concentration, we applied this fraction to a carboxymethyl (CM) Sepharose cation exchange column. The FT fraction (50 mM NaCl) was collected along with 100 mM NaCl, 250 mM NaCl, 500 mM NaCl and 1.0 M NaCl step elution fractions. Again, all of the chromatin assembly activity was found in the FT fraction (Figure 3A). The material that flowed through the DEAE Sepharose and CM Sepharose was then applied to a heparin Sepharose column where the chromatin assembly activity was also found in the FT fraction (Figure 3B).

**Figure 4:**
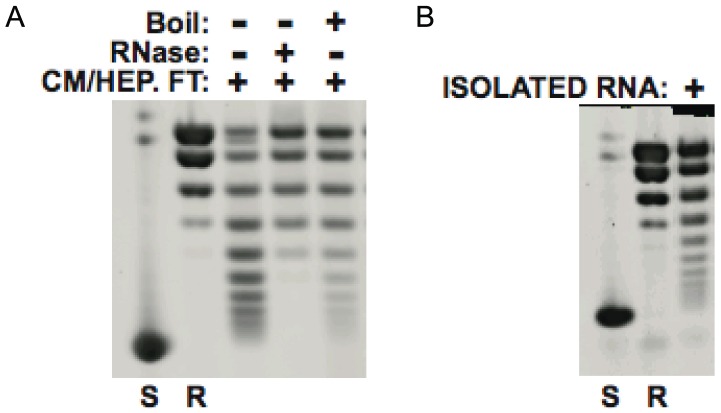
The cytosolic chromatin assembly activity is RNA-dependent. (A) Partially purified chromatin assembly activity (FT from DEAE, CM and heparin sepharose columns) was treated with RNase or boiled as indicated. Sample was then assayed for in vitro chromatin assembly activity. (B) Partially purified assembly activity was extracted with phenol/chloroform and nucleic acids isolated from the aqueous phase by ethanol precipitation. Recovered nucleic acids were assayed for chromatin assembly activity as described above.

The observation that the chromatin assembly activity in the yeast cytosolic extracts did not bind to either DEAE Sepharose, CM Sepharose or Heparin Sepharose columns in low salt buffer was somewhat unusual behavior for a protein. To confirm that the chromatin assembly activity was protein-based, the material that had passed through the DEAE, CM and heparin columns was boiled and the denatured proteins removed by centrifugation. Surprisingly, this had little effect on the chromatin assembly activity (Figure 4A). To determine whether the chromatin assembly activity was dependent on RNA, this material was treated with RNase. The RNase treatment completely eliminated the activity of this fraction (Figure 4A). To confirm the involvement of RNA in the chromatin assembly activity, this fraction was phenol/chloroform extracted multiple times and then nucleic acids were precipitated from the aqueous phase with ethanol. The nucleic acids were then resuspended and assayed for chromatin assembly activity in the presence of rHif1p. Strikingly, the isolated nucleic acids retained a high level of activity (Figure 4B).

### Characterization of the RNA-mediated chromatin assembly activity

**Figure 5:**
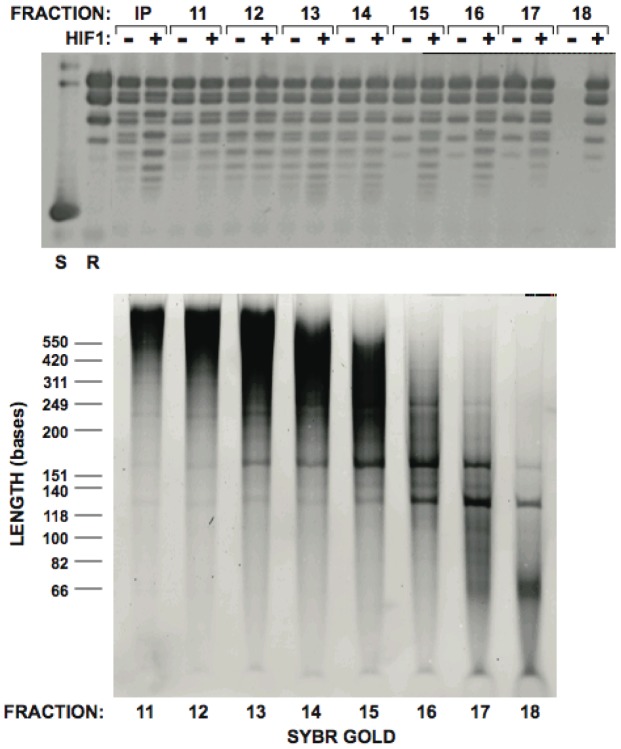
Purification of an RNA-dependent chromatin assembly activity. Cytosolic extract was passed through DEAE, CM and heparin sepharose columns as described above. The partially purified fraction was boiled and clarified by centrifugation. The supernatant was resolved by gel filtration chromatography. Indicated fractions were phenol/chloroform extracted and nucleic acids recovered by ethanol precipitation. Isolated nucleic acids were assayed for chromatin assembly activity in the presence and absence of rHif1p (top). Isolated nucleic acids from the indicated fractions were resolved on a polyacrylamide/urea gel and visualized by Sybr Gold staining (bottom).

The results shown in Figure 4 suggested that RNA was responsible for the chromatin assembly activity that functions with Hif1p *in vitro*. A crucial question to address was whether this was an effect of bulk RNA or whether specific species of RNA were responsible for the chromatin assembly activity. To address this issue, we determined whether the RNA-mediated chromatin assembly activity fractionated as a discreet entity or whether it co-eluted with bulk RNA. The material that flowed-through the DEAE, CM and heparin columns was briefly incubated at 95 degrees and denatured proteins were removed by centrifugation. The soluble fraction was then resolved on a Superose 6 column. Fractions from the column were then phenol/chloroform extracted and nucleic acids were isolated by precipitation. The nucleic acids were then resuspended and assayed for chromatin assembly activity in both the absence and presence of rHif1p. As seen in Figure 5A, a clear peak of chromatin assembly activity is seen from fractions 13–17 and this activity is sensitive to the presence of rHif1p (note that all of the fractions from the column were assayed but only the fractions shown in Figure 5A displayed any activity). To determine the pattern of RNA that eluted from the Superose 6 column, the isolated nucleic acid from each fraction was resolved on a polyacrylamide:urea gel and stained with Sybr Gold nucleic acid stain (Figure 5B, note that all of the species visualized here correspond to RNA as treatment of these fractions with RNase completely eliminates all staining on the gel, data not shown). The most intense staining is found in a large diffuse band of high molecular weight RNA that was most abundant in fractions 11–15. The chromatin assembly activity clearly did not co-elute with this group of RNAs. There were also a number of lower abundance RNAs that elute from the Superose 6 column as discreet species. Most of these RNAs also do not co-elute with the chromatin assembly activity. Hence, these results indicated that the RNA-dependent chromatin assembly activity did not correspond to simply non-specific bulk RNA and suggested that a specific RNA (or group of RNAs) may be involved.

**Figure 6:**
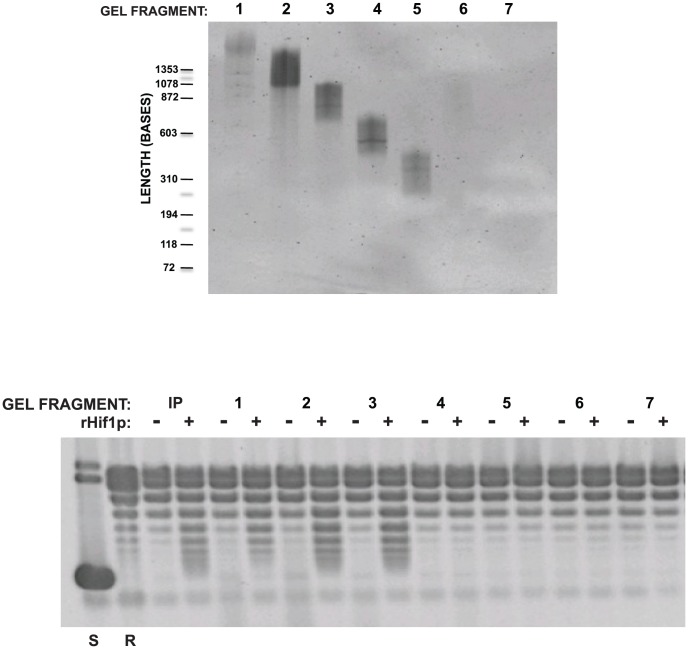
RNA-based chromatin assembly factor localizes to a discreet size range. The material that flowed through the DEAE, carboxymethyl and heparin columns was deproteinized and resolved on a polyacrylamide/urea gel. Segments were cut out of the gel and RNA was eluted (numbered lanes). Recovered RNA was either resolved by polyacrylamide:urea electrophoresis and visualized with Sybr Gold (top) or assayed for chromatin assembly activity with a relaxed circular plasmid, core histones and in the absence or presence of rHif1p, as indicated (bottom). Supercoiled and relaxed plasmid is indicated (S and R, respectively).

To further test whether the chromatin assembly activity was due to the presence of specific RNA species or whether it was attributable to a bulk RNA effect, the isolated RNA that flowed through the DEAE, CM and heparin columns was resolved by polyacrylamide:urea electrophoresis. The gel lane was then cut into a series of 1.0 cm sections and the RNA was eluted from each. Aliquots from each pool of RNA were then either re-run on a gel or assayed for chromatin assembly activity. As seen in Figure 6, the chromatin assembly activity was found in the first three segment containing RNA molecules longer than ∼700 bases. Importantly, the level of chromatin assembly activity did not correlate with bulk RNA concentration as would be predicted if the RNA was functioning as a non-specific polyanion.

### The RNA-mediated chromatin assembly activity is sensitive to histone chaperone mutations

As is the case for most histone chaperones, Hif1p is an acidic protein that contains a large domain that is highly enriched in glutamic acid and aspartic acid residues. Hence, the increased level of chromatin assembly seen when Hif1p is added to the cytosolic extract (or isolated RNA) may simply be a function of Hif1p providing additional negative charge. To test this electrostatic model, we used the human member of the N1/N2 family of histone chaperones, Nuclear Antigenic Sperm Protein (NASP)[17]. NASP is found as two differentially expressed isoforms, tNASP and sNASP[17], [18]. It was previously demonstrated that sNASP is functionally related to Hif1p in that sNASP is capable of stimulating the yeast cytosolic extract chromatin assembly activity[19].

**Figure 7:**
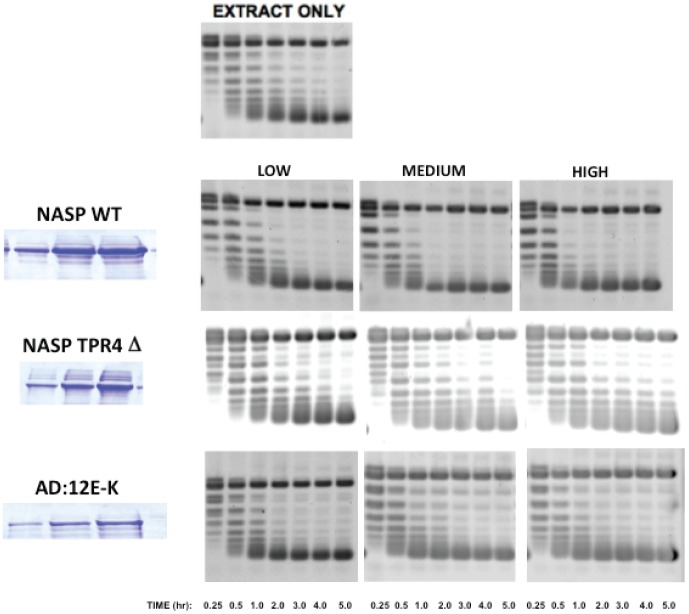
The TPR4 domain of sNASP is important for its activity in RNA-mediated chromatin assembly factors. Chromatin assembly assays were performed with a constant amount of yeast cytosolic extract as described in the legend to Figure 1 except that aliquots of the reactions were taken at 15′, 30′, 1 hr, 2 hr, 3 hr, 4 hr and 5 hr (left to right on each gel). The gel in the upper left hand corner was performed with extract alone. The gels in each row represent reactions performed with 3 concentrations of the indicated form of NASP (WT = wild type, TRP 4Δ = deletion of TPR4 and 12E/K is E to K mutations in the acidic domain of sNASP). The concentrations of NASP in each reaction are indicated by the coomassie blue stained bands on the left of each row. All of the proteins were run on the same gel.

NASP was originally identified as a histone H1 chaperone but was subsequently shown to also interact specifically with H3/H4 complexes[19], [20]. We recently performed a mutational analysis of sNASP and identified distinct domains that are involved in core histone and linker histone binding[21]. This analysis showed that mutations that altered the charge of the acidic domain (sNASP 12E/K) blocked histone H1 binding but did not affect H3/H4 binding. Conversely, deletion of the fourth TPR repeat (TPR 4Δ) of sNASP eliminated core histone binding but not linker histone binding. Importantly, the TPR 4Δ mutation does not decrease the overall negative charge if sNASP. Therefore, we tested whether the TPR 4Δ and 12E/K mutants of sNASP were able to enhance the chromatin assembly activity of the yeast cytosolic extract. Rather than using a single time point, aliquots of the assays taken at various time points were analyzed. Also, we performed the assays with increasing concentrations of each of the full length, TPR 4Δ and 12E/K sNASP constructs. As seen in Figure 7, the cytosolic extract alone was capable of fully assembling nucleosomes on a circular plasmid in 4–5 hours. However, when full-length sNASP was added the reaction proceeded much more rapidly, going to completion within 1 hour. In addition, the effect of full-length sNASP was concentration dependent (most easily seen at the early time points). Strikingly, the fourth TRP repeat was essential for activity of sNASP in these chromatin assembly assays, as the TPR 4Δ mutant appeared to be non-functional. In addition, the sNASP 12 E/K mutant retained an intermediate level of activity. Therefore, these results indicate that the activity of sNASP in histone deposition is not simply to function as a polyanion.

## Discussion

In 1981, Brutlag and colleagues reported that RNA can mediate chromatin assembly[22]. In this publication, the chromatin assembly activity of a Drosophila embryo extract was demonstrated to be sensitive to RNase treatment but resistant to phenol extraction indicating that RNA was the active component in the extract. This observation was followed up with a number of experiments. First, the chromatin assembly activity in the embryo extracts was fractionated on a DEAE cellulose column and was shown to bind strongly to this anion exchange column as would be expected for bulk RNA. Second, a number of natural and artificial polyanions were tested for chromatin assembly activity. Cytoplasmic RNAs isolated from several different cell types (including yeast) had moderate levels of chromatin assembly activity while various preparations of DNA (either single or double-stranded) had no activity. Interestingly, some polyribonucleic acids, such as poly(rC) and tRNA had no chromatin assembly activity while others, such as poly(rU) and poly(rA) were only active when present in vast excess over the histones and DNA in the reaction. These results indicated that, while some RNAs can clearly function in chromatin assembly assays, there is some specificity to this activity and not all polyanions can function in these assays[22]. However, this work appeared at approximately the same time that it was discovered that polyanions such as poly(glutamic acid) could function as a chromatin assembly factor and that, in fact, high levels of salt could also function in this capacity and allow formation of nucleosomes as the salt is gradually dialyzed away[23]. Therefore, the prevailing view became that RNA can function as a non-specific polyanion to facilitate chromatin assembly.

Based on our results, we would like to argue that this prevailing view should be challenged and that the possibility that specific RNA molecules can function in the cell to mediate chromatin assembly should be considered. A number of observations indicate that the RNA-dependent chromatin assembly activity that we have detected is not due to a non-specific effect of bulk RNA. First, unlike the RNA-dependent activity from Drosophila embryos, the activity that we have isolated does not bind to a DEAE column at low salt, as would be expected for the majority of RNA in a cytosolic extract. Second, a highly purified sample of this activity resolves on a gel filtration column as a distinct entity. The fact that the peak of this activity did not coincide with the elution pattern of bulk RNA strongly argues that a specific RNA (or group of RNAs) is capable of functioning as a chromatin assembly factor. Third, the RNA-dependent activity that we have isolated can function in conjunction with protein-based histone chaperones. While the RNA-based activity could generate nucleosomes on its own, the rate at which nucleosomes are formed is significantly enhanced by the presence of the histone chaperones. Importantly, the ability of histone chaperones to function with this RNA-based activity is sensitive to mutations in the histone chaperones that do not alter their electrostatic properties. Finally, our understanding of the breadth of functions that are performed in the cell by RNA has exploded in the decades since RNA was first shown to mediate chromatin assembly in vitro. For example, it is now abundantly clear that specific RNA molecules play a critical role in the targeting and function of chromatin modifying factors at specific sites in the genome[24]–[31]. Hence, the proposition that RNA can play a specific role in chromatin assembly may not be as far-fetched now as it would have been 30 years ago.

## Materials and Methods

### Yeast cytosolic extract preparation

Yeast strain XAY4 (Hif1Δ) was grown to mid-log phase (OD_600_∼0.5) and harvested by centrifugation (∼4000×g for 5 minutes)[10]. Spheroplasts were generated from freshly harvested cells as described previously[32]. Spheroplasts were lysed by the addition of one volume lysis buffer (10 mM HEPES, 18% W/V Ficoll 400) and then diluted with two volumes of buffer A (10 mM HEPES [pH 6.0], 50 mM NaCl, 1 mM MgCl_2_). Lysed spheroplasts were centrifuged at 1500×g for 15 minutes to pellet nuclei and cell debris and the supernatant used as the cytosolic extract[33].

### Recombinant protein production

Recombinant Hif1p was produced as described[10]. Full length and TPR4D sNASP expressed and isolated as described previously[21].

### Chromatin assembly assays

Supercoiled pUC18 plasmid DNA was isolated from E. coli and relaxed with topoisomerase I using manufacturers instructions (Sigma). In vitro chromatin assembly assays were performed in a total volume of 25 µL. The assay buffer contained 10 mM Tris (pH8.0), 1 mM EDTA, 100 mM NaCl and 100 µg BSA. Reactions also contained 0.2 µg relaxed pUC18 DNA, 0.5 to 1.0 µg purified chicken erythrocyte histones and rHif1p and yeast cytosolic extract or column fraction as appropriate. Reactions were incubated at 37 degrees for 1 hour and then stopped by the addition of 6.25 mL of 5× stop buffer (1% SDS, 500 µg/mL Proteinase K) and an additional 30 minute incubation at 37 degrees. DNA from each reaction was then isolated by phenol/chloroform extraction followed by ethanol precipitation. DNA was resolved on a 1.5% agarose gel run overnight at 12V. DNA was then visualized by staining with Sybr Gold.

### Column chromatography

Cytosolic extract was diluted with DN(0) buffer to match the conductivity of DN(950) buffer and then clarified by centrifugation at 16,000×g for 10 minutes (DN buffers contain 25 mM Tris [pH 7.0], 0.1 mM EDTA, 10% glycerol and the concentration of NaCl, in mM, given in parentheses). Clarified extract was applied to 25 mL DEAE Sepharose column (using an AKTA Purifier) and the column was extensively washed with DN(50). The column was then successively step eluted with DN(100), DN(250) and DN(500). Material that flowed through the DEAE column (FT) was dialyzed against 50 mM HEPES (pH 6.0), 50 mM NaCl buffer and applied to a 5 mL carboxymethyl Sepharose column (CM). After washing, the CM column was successively eluted with buffer containing 100 mM, 250 mM, 500 mM and 1 M NaCl. The FT from the CM column was directly applied to a 1 mL Heparin Sepharose column. Isolated RNA was resuspended in TE and 250 µL was applied to a 25 mL Superose 6 column, which was run at 0.3 mL per minute and 1 mL fractions were collected.

### RNA isolation

RNA was isolated from extracts or column fractions by phenol/chloroform extraction followed by ethanol precipitation. RNA was isolated from gels following electrophoresis of RNA samples on a 6% polyacrylamide/8M urea gel. Gels were visualized with Sybr Gold. Gel slices were excised with a razor blade and crushed in a microfuge tube. Gel pieces were incubated with 1.0 mL of 0.3 M NaCl overnight at 4 degrees on a rotator. Supernatant was separated from the gel pieces and RNA precipitated with isopropyl alcohol.
